# Reemergence of Cholera in Haiti

**DOI:** 10.1056/NEJMc2213908

**Published:** 2022-11-30

**Authors:** Daniel H. F. Rubin, Franz G. Zingl, Deborah R. Leitner, Ralph Ternier, Valusnor Compere, Samson Marseille, Damien Slater, Jason B. Harris, Fahima Chowdhury, Firdausi Qadri, Jacques Boncy, Louise C. Ivers, Matthew K. Waldor

**Affiliations:** Brigham and Women’s Hospital Boston, MA; Brigham and Women’s Hospital Boston, MA; Brigham and Women’s Hospital Boston, MA; Zanmi Lasante Croix-des-Bouquets, Haiti; Laboratoire National de Santé Publique Port-au-Prince, Haiti; Laboratoire National de Santé Publique Port-au-Prince, Haiti; Massachusetts General Hospital Boston, MA; Massachusetts General Hospital Boston, MA; International Centre for Diarrhoeal Disease Research, Bangladesh (ICDDR,B) Dhaka, Bangladesh; International Centre for Diarrhoeal Disease Research, Bangladesh (ICDDR,B) Dhaka, Bangladesh; Laboratoire National de Santé Publique Port-au-Prince, Haiti; Massachusetts General Hospital Boston, MA; Brigham and Women’s Hospital Boston, MA

## To the Editor:

Cholera was absent from Haiti until an inadvertent introduction by United Nations security forces in October 2010. The ensuing epidemic sickened 820,000 and caused 9,792 reported deaths^1^. The last cholera case in Haiti was recorded in January 2019, and in February 2022, Haiti was declared to have eliminated cholera^2^. In late September of 2022, a new outbreak began in Port-au-Prince and rapidly expanded to 9,317 suspected cases by mid-November of which >800 were confirmed by culture^3^. Here, we present genomic and phenotypic analysis of the *Vibrio cholerae* isolated from a stool sample collected on September 30th, 2022 of an index case – a child who presented with watery diarrhea and severe dehydration – to begin to address the origins of the epidemic.

The 2022 *V. cholerae* isolate shares phenotypes with the 2010 outbreak strain. Both strains are *V. cholerae* serogroup O1 of the Ogawa serotype and have similar antibiograms, including resistance to trimethoprim/sulfamethoxazole and low-level resistance to ciprofloxacin (Supp. Table 1,2). This resistance profile is consistent among 130 isolates from the current outbreak, suggesting that the strain isolated from the index case is representative of the ongoing epidemic.

To decipher the relationship between the current outbreak strain and other toxigenic O1 El Tor strains from the ongoing seventh pandemic of cholera, we sequenced the 9/30/2022 isolate, along with four 2021-2022 isolates from Dhaka, Bangladesh (Supp. Table 2). Phylogenetic analyses of >1,200 isolates revealed that the 2022 Haiti isolate is closely related to 2010 Nepal isolates that were the origin of the initial outbreak. The 2022 isolate belongs to a subclade of Haiti *V. cholerae* isolates originating in 2013 during the previous epidemic and is divergent from 2013 strains from Mexico that were thought to have spread from Haiti, as well as currently circulating Bangladesh isolates. Haiti 2022 and Haiti 2010 isolates have identical *ctxB* (*ctxB7*) and other virulence factors (Supp. Table 3) and produce similar quantities of cholera toxin (Supp. Figure 1).

These analyses suggest that the reemergence of cholera in Haiti is caused, at least in part, by a descendant of the *V. cholerae* strain that gave rise to the 2010 epidemic. However, no cases of cholera were confirmed between 2019 and 2022 despite ongoing surveillance. Several explanations for the recrudescence of this strain are possible. The first is that toxigenic *V. cholerae* O1 persisted in Haiti through sub-clinical human infection and has recurred in the setting of waning population immunity coupled with a crisis in lack of clean water and sanitation. Another non-exclusive possibility is that this *V. cholerae* strain has persisted in environmental reservoirs. Finally, since the Haiti outbreak was ultimately transmitted to other countries in Latin America^4^, a third less likely explanation, given the phylogenetic evidence and absence of recent cholera cases in the region, is that the current strain could have been reintroduced to Haiti from a nearby country. These findings, along with the resurgence of cholera in several parts of the world^5^ despite available tools, suggest that cholera control and prevention efforts must be redoubled.

## Supplementary Material

supplement

## Figures and Tables

**Figure 1.**
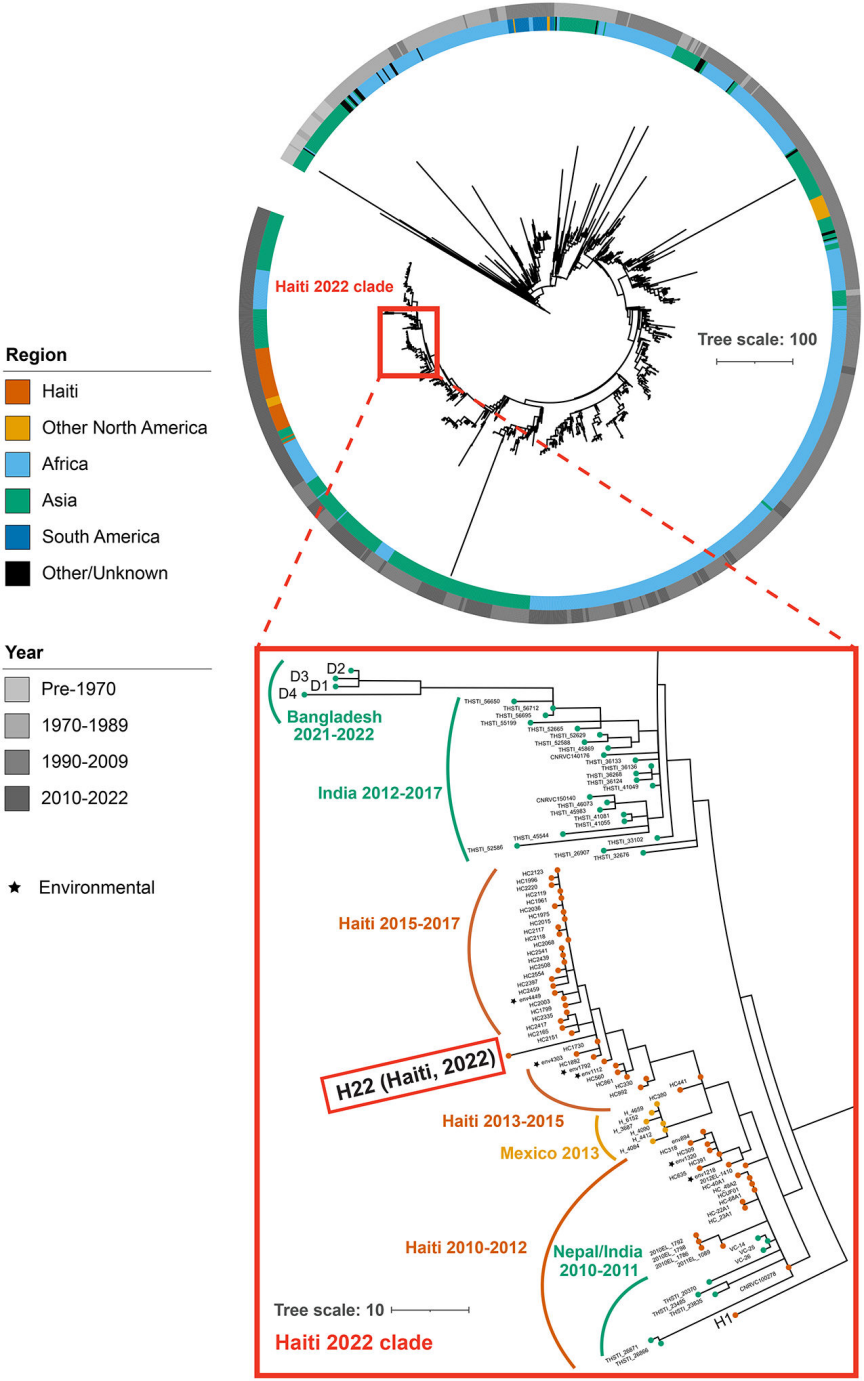
Phylogenetic tree of 7^th^ pandemic *Vibrio cholerae*. (Top) A phylogenetic tree of non-recombinogenic regions from 1,270 strains of O1 El Tor 7^th^ pandemic *V. cholerae*. Tracks represent continent (inner) and year (outer) of isolation. Tree scale represents single-nucleotide polymorphisms (SNPs) per genome. (Bottom) Inset focused on the Haiti clade along with recent Asian isolates.
